# Structural Characterization of H1N1 Nucleoprotein-Nucleozin Binding Sites

**DOI:** 10.1038/srep29684

**Published:** 2016-07-11

**Authors:** Bo Pang, Nam Nam Cheung, Weizhe Zhang, Jun Dai, Richard Y. Kao, Hongmin Zhang, Quan Hao

**Affiliations:** 1 School of Biomedical Sciences, The University of Hong Kong, Hong Kong SAR, China; 2 Department of Microbiology, The University of Hong Kong, Hong Kong SAR, China; 3 Research Centre of Infection and Immunity, The University of Hong Kong, Hong Kong SAR, China; 4 Carol Yu Centre for Infection, Li Ka Shing Faculty of Medicine, The University of Hong Kong, Hong Kong SAR, China; 5 State Key Laboratory of Emerging Infectious Diseases, Hong Kong SAR, China; 6 Department of Biology and Shenzhen Key Laboratory of Cell Microenvironment, Southern University of Science and Technology, Shenzhen 518055, China

## Abstract

Influenza viruses are among the most common pathogens that threaten the health of humans and animals worldwide. Various anti-viral therapeutic agents are currently used for treatment and prophylaxis of influenza virus, but the targets of these drugs are easily mutated and result in resistance. Therefore, medications that have broad spectrum coverage are urgently needed to combat with the disease. Since nucleoprotein is regarded as a druggable target due to its conserved sequence and important functions during influenza virus life cycle, numerous studies are focused on this protein in attempts to develop broad-spectrum anti-influenza therapeutics. Recently, a novel small molecule compound, nucleozin, was found to induce large aggregates of nucleoprotein, which in turn caused cessation of virus replication. However, the aggregation-inducing mechanism of nucleozin has not been unveiled. Here we report the crystal structure of nucleoprotein-nucleozin complex at 3 Å resolution, which shows the binding sites of nucleozin at nucleoprotein for the first time. The complex structure reveals how nucleoprotein and nucleozin interact with each other and hence result in nucleoprotein aggregates. The structural information is envisaged to help accelerate the development of anti-influenza therapeutic agents.

Although influenza is usually acute self-limiting respiratory infection, influenza viruses are among the most common pathogens that threaten the health of humans and animals worldwide. Every year, seasonal influenza would cause 250,000–500,000 deaths worldwide. Currently, two classes of anti-influenza drugs are available on the market, M2 proton channel blockers and neuraminidase inhibitors. However, M2 ion channel blockers, amantadine and rimantadine, are no longer recommended for clinical treatment of influenza A virus infection due to their wide spread of drug resistance. After four decades of effective usage, amantadine resistant influenza A viruses currently have reached or exceeded 90% in the United States. In the 2008/2009 flu season, the US CDC identified that 100% of seasonal H3N2 and 2009 pandemic H1N1 flu samples were resistant to both amantadine and rimantadine^1^,^2^,^3^,^4^. Similarly, influenza viruses resistant to neuraminidase inhibitors are increasingly reported in recent years. Resistance to neurominidase inhibitor oseltamivir was found in influenza strain H3N2^5^ in 2004 and avian H5N1 strain^6^ in 2005. Furthermore, almost all circulating seasonal H1N1 strains in 2008–2009 and 1% of tested 2009 pandemic H1N1 viruses were resistant to oseltamivir^7^. Therefore, discovery of novel agents for prevention and treatment of influenza is imperative and urgently needed.

Influenza A virus belongs to the orthomyxoviridae family^8^. The central core of influenza virus contains the genome composed of eight fragments of negative-sense single strand RNA and each of them encodes one or two proteins^9^. In total, the genome of influenza A virus encodes eleven proteins, i.e. haemagglutinin (HA), neuraminidase (NA), nucleoprotein (NP), matrix protein (M1 and M2), non-structure proteins (NS1 and NS2) and polymerase (PA, PB1, PB1- F2 and PB2)^10^. Among them, NP is regarded as an attractive target due to its highly conservative and multifunctional nature during virus life cycle. In addition, NP does not have any counterpart in the cells, and hence compounds targeting NP might have less off-target side effects^11^,^12^.

Interestingly, Kao *et al*. identified NP as an anti-influenza drug target using advanced chemical genetics approach, and reported that a novel small molecule named nucleozin could target this protein^13^. NP forms large aggregates in the presence of nucleozin, and is restrained from nuclear accumulation, which in turn results in cessation of virus replication. Later, Gerritz *et al*. conducted similar research on nucleozin and its analogs, showing that nucleozin and its analogs exert certain inhibitory effect on influenza virus life cycle^14^. In addition, they also attained the crystal structure of NP in complex with a nucleozin analog through co-crystallization, and found that the binding sites of the nucleozin analog (Compound 3) were formed by a combination of the pockets from two different NP trimers, i.e., Y289/N309 pocket from one trimer and Y52 pocket from the other one. However, the binding mode revealed in this structure can only induce small oligomers of NP (dimer of trimers) and cannot explain the large NP aggregates. Here we report the crystal structure of NP-nucleozin complex at 3 Å resolution. The binding sites have been clearly revealed and the binding model of nucleozin induced NP aggregates is proposed, which may not only help to understand the unique mechanism of nucleozin-induced NP aggregation at atomic level, but also shed new light on discovery of novel drugs targeting NP.

## Results

### X-Ray crystal structure of nucleozin bound NP reveals a protein dimer of trimers

**Figure 1:**
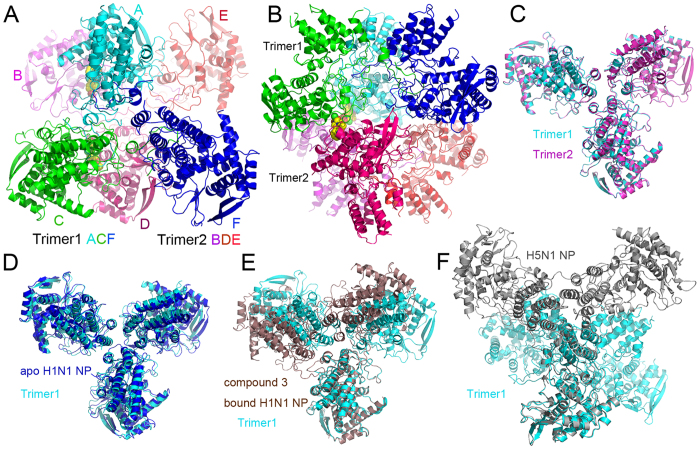
H1N1 NP-nucleozin complex in the asymmetric unit and comparison with other NP structures. There were six NP molecules (molecules A–F) in the asymmetric unit and they were coloured in cyan (A), magenta (B) green (C), pink (D), red (E) and blue (F) in panels A and B. These six molecules formed dimer of trimers. Trimer 1 was composed of chains A, C and F while trimer 2 was composed of chains B, D and E. Two nucleozin molecules were found between chains A and B, C and D, respectively. They were shown in space-filling model with yellow carbon. (**A**) Top view of dimer of trimers. (**B**) Side view of dimer of trimers. The two trimers in the asymmetric unit can be superimposed perfectly with an r.m.s.d. value of 0.28 Å (**C**). The trimer was superimposed to apo H1N1 NP trimer (D, blue), Compound 3 bound H1N1 NP (E, brown) and H5N1 NP (**F**, grey). It is obvious that the NP-nucleozin structure was very close to apo H1N1 NP, but not compound 3 bound NP nor H5N1 NP.

The crystal structure of nucleozin bound H1N1 NP has been solved at a resolution of 3.0 Å and there are six NP molecules assembled into two trimers in the crystallographic asymmetric unit (Fig. 1A,B). Similar to the reported apo H1N1 NP^15^ (PDB code 2IQH) and H5N1 NP^16^ (PDB code 2Q06), each NP molecule in the asymmetric unit folds into three parts, a helical body domain, a helical head domain and a tail loop inserted into its neighboring molecule to form NP trimers. The three NP molecules in each trimer in the current structure are not identical with Cα superposition r.m.s.d. values between 0.55 and 1.19 Å. This moderate similarity is also found when comparing current NP structures with the apo H1N1 NP (r.m.s.d. values between 0.75 and 1.37 Å), H5N1 NP (0.69~0.77 Å) and Compound 3 bound H1N1 NP^14^ (PDB code 3RO5, 0.61~1.46 Å). However, the two trimers in the asymmetric unit are almost identical with an r.m.s.d. value of 0.28 Å (Fig. 1C). The trimers are similar to the apo H1N1 NP trimer with an r.m.s.d. value of 2.07 Å (Fig. 1D), but are in sharp contrast to the H5N1 NP and Compound 3 bound H1N1 NP (Fig. 1F,E). As suggested in the H5N1 structure, the intrinsic flexibility of the tail loop may contribute to the formation of various NP trimers to support its multifunctions in viral life cycle. The variation of trimer formation also provides clues for high-order oligomer formation. The interfacial area calculated by PISA^17^ is 630.4 Å^2^ between Chain A and B, ΔG is 1.6 kcal/mol, and potential number of hydrogen bond (Nhb) is 10; while between Chain C and D, these values are 654.1 Å^2^, 1.3 kcal/mol and 10, respectively. In the current structure, two nucleozin compounds were found to bridge the two trimers in the asymmetric unit forming a dimer of trimers. Although the contact area between the two trimers is not large, nucleozin was found to provide pivotal clues for high-order NP aggregate formation which will be further discussed later.

### Nucleozin binding in NP

**Figure 2:**
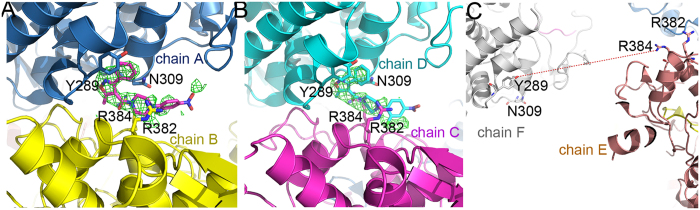
Two nucleozin molecules found in the asymmetric unit. NP molecules were shown as carton while nucleozin and key interaction residues were shown as stick models. Nulceozin omit map was shown as green mash at 2σ. (**A**) Nucleozin between chains A and B; (**B**) Nucleozin between chains C and D; (**C**) No nucleozin found between chains F and E. The Y289/N309 pocket in chain F was about 40 Å away (red dashed line) from the R383/R384 pocket in chain E.

**Figure 3:**
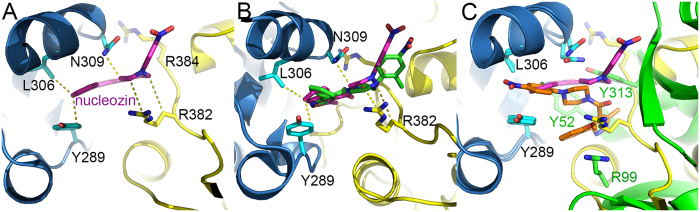
Detailed view of the interaction between nucleozin and NP. The two NP chains interacting with nucleozin or compound 3 were shown as cartoon and coloured in blue and yellow or blue and green, respectively. Residues interacting with nucleozin or compound 3 (Y289, L306, N309, R382, R384, Y52, Y313 and R99) and the two compounds (nucleozin and compound 3) were shown as stick models. (**A**) Key NP-nucleozin interactions in one NP trimer were shown as dashed lines. Nucleozin was shown with magenta carbons. (**B**) Comparison of the two nucleozin molecules (magenta and green) in the asymmetric unit. The two NP-nucleozin complexes were superimposed and key interactions between nucleozin and NP were shown as dashed lines. (**C**) Structural comparison between nucleozin-bound and Compound 3 bound NP. Nucleozin was shown with magenta carbons and Compound 3 with brown carbons. Key interaction residues were shown as stick models and labelled.

There are six NP molecules in the asymmetric unit while only two nucleozin molecules were identified in the structure (Fig. 2). It is very clear that one nucleozin molecule bridges two NP molecules in two adjacent trimers, and the binding pockets are formed by combination of the Y289/N309 pocket from one NP molecule and R382 pocket from the other NP molecule. The Y289/N309 pocket is composed of hydrophobic interactions with side chains of residues Y289, F291, L306, R305 and N309 (Fig. 3A,B). In this pocket, the aromatic ring of residue Y289 forms hydrophobic interactions with the phenyl ring part of nucleozin through π stacking. Residue N309 forms both hydrophobic and weak hydrogen bond interactions with the middle part of nucleozin. The R382 pocket, composed of residues R382 and R384, supports the nitro-phenyl moiety of nucleozin like a horse saddle. These two residues interact with nucleozin through weak polar interactions. Although the interactions between the R382 pocket and nucleozin are not very strong, it is vital in nucleozin binding with NP. In our crystal structure, only two nucleozin molecules were identified in six NP chains. In the NP-nucleozin complex structure, nucleozin molecules only bind with Y289/N309 pockets from Chain A and Chain D (Fig. 2), and it has not been found in the other four Y289/N309 pockets and none of the Y52/Y313 pockets as revealed in the nucleozin analog-NP structure. The underlying reasons might be that, the interaction between nucleozin and Y289/N309 pocket is essential but not enough for their binding, so only these two Y289/N309 pockets in Chain A and D which get additional support from the R382 pocket in an adjacent NP chain are able to stabilize nucleozin molecules. On the contrary, the other Y289/N309 pockets and Y52/Y313 pockets did not get extra support from nearby binding pocket and hence no nucleozin was found there, suggesting that the R382 pocket is also important to help nucleozin binding.

Similar to nucleozin binding in NP, the binding of nucleozin analog Compound 3 in NP is also composed of two binding pockets^14^, the Y289/N309 pocket and the Y52 pocket. Of these two pockets, the Y289/N309 pocket is found in both compounds’ binding while the Y52 pocket is not found in the nucleozin binding. In the Y289/N309 binding pocket, in sharp contrast to the nucleozin binding in NP, the binding of Compound 3 in NP is in the reversed conformation (Fig. 3C) where the nitro-phenyl part of Compound 3 forms hydrophobic interactions with the Y289 pocket. And the methoxy phenyl ring part of Compound 3 twisted back and forms hydrophobic interactions with side chains of residues Y289 and R99. Residue Y52 forms hydrophobic interactions with the middle part of Compound 3. The different binding patterns of these two compounds with NP might be due to their different structures and hence the different properties in inducing aggregation where nucleozin can induce large NP aggregates while Compound 3 can only induce NP hexamers.

### Y289/N309 pocket is essential to nucleozin binding

As shown above, the Y289/N309 pocket is common for both nucleozin and compound 3 binding of NP, indicating the importance of this pocket in compound binding. Mutations at this pocket confer virus resistance to nucleozin and are found in both natural circulating viruses (such as H1N1 A/CA/07/2009 (Y289H) pandemic flu and H1N1 A/Solomon Islands/3/2006 (N309T)) and artificially selected viruses (Y289H, N309K) through multiple passages in high concentrations of nucleozin^13^,^14^. As shown in the NP-nucleozin and NP-compound 3 structures (Fig. 3), the phenyl ring of Y289 forms strong π stacking with the compounds. In the Y289H mutant, this kind of interaction will be abolished, resulting in resistance to these compounds. Although the interaction between nucleozin and residue N309 is weak from the structural view (Fig. 3), it is obvious that N309K mutant would interfere the binding of nucleozin into the Y289 pocket through physical obstacle. The longer side chain of lysine would protrude towards nucleozin and hence block the binding. As to the N309T mutant, although it is small and cannot perturb the binding like N309K, it lacks hydrogen bonding with the compound compared with N309, resulting in moderate resistance to nucleozin^14^.

### R382 is involved in nucleozin induced NP aggregation

**Figure 4:**
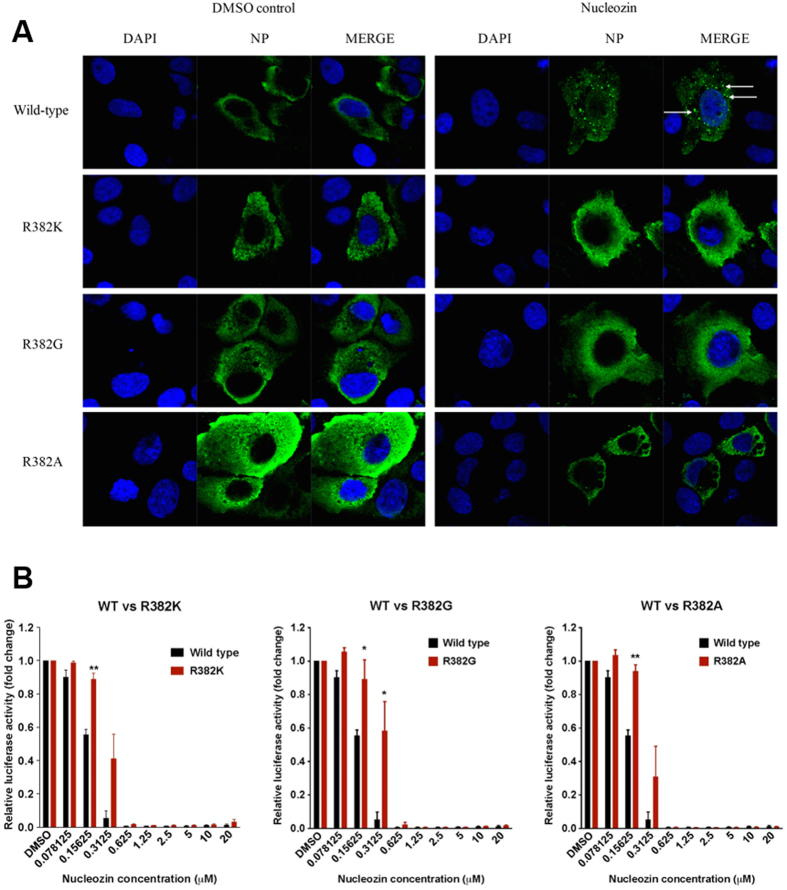
Amino acid substitutions at R382 interfere with nucleozin activities. (**A**) MDCK cells transfected with a plasmid encoding wild-type or mutant NP were treated with DMSO or 1 μM of nucleozin for 8 hrs. DAPI staining and mouse anti-influenza A NP antibodies were used to define the locations of the nucleus and NP respectively. White arrows indicated the aggregated NP. (**B**) Luciferase-based minigenome assay. HEK293T cells were transfected with multiple plasmids encoding each of the following: firefly-luciferase reporter, renilla control, influenza A/WSN/33 PA, PB1, PB2 and either wild-type NP or NP containing the indicated mutations. Serial dilution of nucleozin was added 2 hours post-transfection and the luciferase activity was measured on the next day. The mean ± SD shown here were of three independent experiments performed in triplicates. *P < 0.05; **P < 0.01.

To investigate if residue R382 could be involved in nucleozin induced NP aggregation, we expressed influenza A/WSN/33 (H1N1) wild-type NP or R382 mutant NP in MDCK cells with the addition of 1 μM nucleozin or DMSO (vehicle control). The formation of NP aggregates could be readily observed in cells transfected with wild-type NP 8 hours after the addition of nucleozin, while no aggregates formed in cells transfected with R382 mutant NP or DMSO treated samples (Fig. 4A). This assay strongly supports that aggregation of NP is induced by nucleozin and residue R382 participates the process of nucleozin induced NP aggregation.

The role of R382 was further tested using a luciferase reporter–based mini-genome assay, which measures the transcription and replication activity of the viral polymerase. HEK293T cells were transfected with plasmids encoding the viral polymerases as well as a luciferase reporter and a renilla control. Concentrations at which nucleozin still effectively abolished viral polymerase activity in wild-type NP, had minimal effect on the mutated NP (Fig. 4B). These cellular assays highlight the importance of residue R382 in the nucleozin induced inhibition of NP function.

## Discussion

In previous research, Kao *et al*. predicted the binding mode of nucleozin in NP based on computational model^13^. However, this model cannot explain how nucleozin induces the oligomerization of NP molecules. And later in the nucleozin analog-NP complex structure, two compounds bridge two NP molecules bottom-to-bottom through the Y289/N309 and Y52/Y313 pockets. Through crystallographic three-fold symmetry, NP molecules form a closed hexamer and no more NP molecules can be added to this complex to form large aggregates. In contrast, our current research provides new insight toward this question by indicating that nucleozin would bind two NP molecules at the same time rather than binding a single NP proposed in the computational predication, and this binding model gives the possibility to form nucleozin-induced large aggregates, which will be discussed later.

The nucleozin binding, as revealed in our structure, needs two binding pockets. The first one is the Y289/N309 pocket. As indicated by Kao *et al*.^13^, residue Y289 is essential for the binding of nucleozin to NP. Y289H mutant of NP is resistant to nucleozin and the mutant NP would enter into the nucleus instead of accumulation in the cytoplasm. Our crystal structure of the NP-nucleozin complex provides clear evidence for these interactions by showing that there is a π-stacking between the aromatic ring of residue Y289 and nucleozin, and N309 forms both hydrophobic and hydrogen bonding interactions with nucleozin, suggesting that Y289/N309 pocket is indispensable to the aggregation process induced by nucleozin.

**Figure 5:**
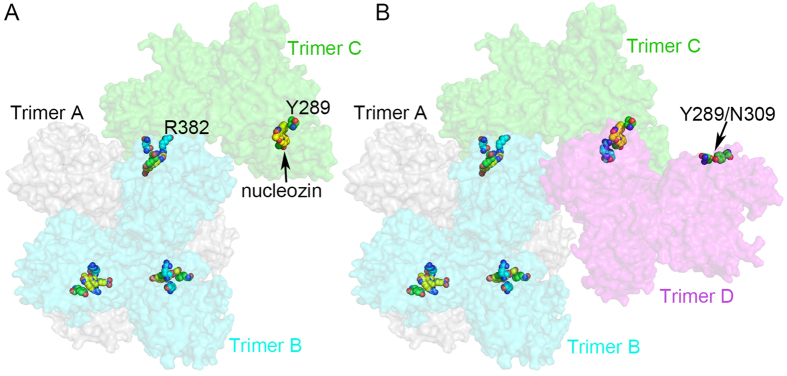
Model for NP-Nucleozin aggregation using the Y289/N309 and R382/R384 pockets. NP trimers were shown as surface representation and colored in grey (trimer A), cyan (trimer B), green (trimer C) and magenta (trimer D). Nucleozin, Y289 pocket and R382 pockets were shown as sphere models with yellow, green and cyan carbons, respectively. (**A**) The docking of trimer C onto the hexamer of NP. There were still nucleozin binding pockets available in trimer C (indicated by black arrow) for the binding of the fourth NP trimer. (**B**) The docking of trimer D onto trimer C. Unoccupied nucleozin binding pocket in trimer D was indicated by a black arrow.

The other binding pocket of nucleozin identified in our structure is the R382/R384 pocket. Although this pocket provides less binding affinity to nucleozin, it is important for nucleozin binding because the Y289/N309 or Y52/Y313 pocket itself cannot bind nucleozin. Based on such observations, a nucleozin induced NP aggregation model has been generated with the help of computational tools. Rosetta docking online software (http://rosettadock.graylab.jhu.edu) has been employed to verify and optimize structure detail of protein-protein interaction between the nucleozin bridged two trimers. As shown in Fig. 5, nucleozin binds one NP molecule from the third trimer (C) through the R382 pocket and one NP molecule in trimer B through Y289/N309 pocket. There are still two NP molecules from trimer C with unbound Y289/N309 and R382 pockets. The fourth trimer (D) could bind trimer C via nucleozin following the same binding property. The results of addition of trimers C and D are shown in Fig. 5. It is obvious that some binding pockets in trimer D are still exposed and could bind other NP trimer. Therefore, in this model, NP molecules could keep on binding each other until there is no binding site exposed.

Although we could not obtain viable viruses with R382 residue mutated to K, G, or A using reverse genetics due to the high possibility that R382 is essential for effective propagation of the virus, we were able to illustrate the involvement of R382 in nucleozin resistance and nucleozin induced NP aggregation by *in vitro* minigenome and immunofluorescence assays. This further supports the biological relevance of our crystal structure of nucleozin-NP complex.

**Figure 6:**
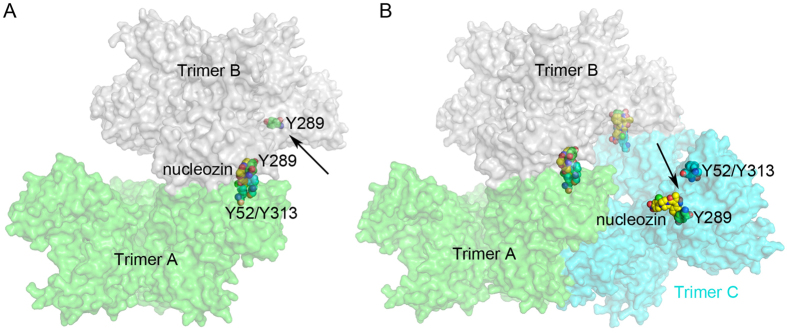
Model for NP-Nucleozin aggregation using the Y289/N309 and Y52/Y313 pockets. NP trimers were shown as surface representation and colored in green (trimer A), grey (trimer B) and cyan (trimer C). Nucleozin, Y289 pocket and R382 pockets were shown as sphere models with yellow, green and cyan carbons, respectively. (**A**) Trimer B docked on trimer A. Unoccupied nucleozin binding pocket in trimer B was indicated by a black arrow. (**B**) Trimer C docked onto trimer B. There were still nucelozin binding sites available in trimer C (indicated by the black arrow).

Because biological data suggest that Y52/Y313 are also important for nucleozin binding and Y52H mutant is resistant to nucleozin^13^, we tried to build a model with the Y289/N309 and Y52/Y313 pockets as nucleozin binding sites. Similar to the above-mentioned process, NP trimer is still used as basic building unit in this modelling process. As shown in Fig. 6, the nitro-phenyl group of nucleozin could bind Y52/Y313 pocket of one NP molecule in trimer A and the phenyl ring part of nucleozin binds the Y289/N309 pocket of the other NP molecule in trimer B. And then another trimer C could bind trimer B via nucleozin following the same procedure. It is obvious that some binding pockets in trimer A, B or C are still exposed and could bind other NP trimers. As a result, in this model, NP molecules could also keep on binding each other to form large NP aggregates.

In summary, the crystal structure of NP-nucleozin complex revealed two binding pockets of nucleozin, which are both vital for the interaction between NP and nucleozin. Based on the structure, we propose that nucleozin could link two NP molecules into dimeric subunits, and then further induce the formation of large aggregates, which is illustrated in our computational models. Moreover, the structural details of the ligand binding sites will certainly provide clues for further modification of drug candidates, which makes it possible to overcome drug resistance through chemical modification of the compounds. In addition, the Y289/N309 and R382/R384 pocket could be treated as a novel drug target, and structure-based screening could also be conducted to identify new ligands that would possibly bind this site with high affinity.

## Materials and Methods

### Cell lines and plasmids

Cell lines were purchased from ATCC and maintained in MEM supplemented with 10% heat inactivated fetal bovine serum (HI-FBS). cDNA of wild-type NP or NP containing mutations R382K, R382G, R382A, four segments making up the viral polymerase of influenza A/WSN/33 (H1N1) was cloned into pHW2000 plasmids (pHW2000-PB2, pHW2000-PB1, pHW2000-PA, pHW2000-NP) and used for the luciferase reporter assay.

### Immunofluorescence microscopy

MDCK cells were seeded at 5 × 10^4^ cells per coverslip one day before transfection of a plasmid encoding the indicated gene using Lipofectamine LTX according to the manufacturer’s instructions. Nucleozin was added on the next day at a concentration of 1 μM. Coverslips were harvested at 8 hours post-drug addition and fixed using 3.7% formaldehyde in PBS for 10 min. Cells were then permeabilized using 0.1% Triton X-100 for 4 min and blocked with 10% FBS in PBS for 30 min before incubation with mouse primary antibody against NP (Millipore) in 1% FBS in PBS for 1 h at 37 °C. Coverslips were then incubated with rabbit secondary antibody against mouse (AF488; Invitrogen) in 1% FBS in PBS for 1 h at 37 °C. Coverslips were placed “face down” onto a drop of Mounting Medium with Dapi (Vectashield) on a microscope slide before sealing of the coverslip with nail polish. Fluorescent images were obtained using a Carl Zeiss LSM710 upright laser scanning confocal microscope with a Plan Apochromat 63 × 1.4 oil immersion objective (The University of Hong Kong Li Ka Shing Faculty of Medicine Faculty Core Facility).

### Minigenome Assay

Full-length genomic segments of influenza A/WSN/33 PA, PB1, PB2, NP or NP mutants were cloned into a pHW2000 vector. The four segments making up the viral polymerase were co-transfected into HEK293T cells together with a luciferase reporter plasmid, which contains a noncoding sequence from the M segment of influenza A virus and the firefly luciferase gene, and a plasmid encoding the renilla control. Two hours after transfection, DMSO (vehicle control) or a serial dilution of nucleozin was added to the transfected cells. Twenty-four hours after transfection, the luciferase activities were measured using Dual Luciferase Assay System kit (Promega E1910) and Victor3 multilabel plate reader (Perkin Elmer).

### Protein expression and purification

The full length sequence was cloned into a pET28 vector (Novagen) with a hexa-histidine tag at its N-terminus to facilitate purification. The recombinant plasmid pET28-NP was transformed into *Escherichia.coli* strain Rosetta 2 and the bacteria were cultured at 37 °C. Protein expression was induced at cell density of OD600 = 0.6 with 0.5 mM IPTG for about 16 hours at 18 °C. The cell pellet was harvest and lysed by sonication in lysis buffer (20 mM HEPES, pH 7.5, 500 mM sodium chloride), and the cell debris was removed by centrifugation.

The cleared lysate was loaded onto a HisTrap HP column (GE Healthcare) mounted on an AKTA purifier (GE Healthcare). The column was washed with 50 ml of lysis buffer and then the protein was eluted with a linear imidazole gradient from 20–200 mM. The NP protein was collected, concentrated and subjected to size exclusion chromatography using a Superdex 200 16/60 column (GE Healthcare) pre-equilibrated with buffer containing 20 mM HEPES, pH 7.5, 150 mM sodium chloride. The purified protein was concentrated to 20 mg/ml for crystallization trials.

### Crystallization, diffraction data collection, and structure refinement

The crystallization screening was conducted by sitting-drop vapor diffusion method. Crystals were obtained in 0.1 M sodium acetate, 0.05 M magnesium acetate, 0.1 M MES, pH 6.0, 7% PEG8000. Crystals of NP-Nucleozin complex were prepared by soaking native crystals in crystallization solution containing 0.25 mM nucleozin for 2 hours, and then the crystals were frozen by quick immersion in liquid nitrogen. Cryoprotectant solution contains 0.1 M sodium acetate, 0.05 M magnesium acetate, 0.1 M MES, pH 6.0, 15% PEG8000, and 20% PEG400.

The X-ray diffraction data were collected at beamline BL17U at the Shanghai Synchrotron Radiation Facility and processed with HKL2000 package^18^. Molecular replacement method was used to solve the structure using program PHASER^19^ in the CCP4 program suite^20^, and the apo H1N1 nucleoprotein structure (PDB code 2IQH)^15^ was used as a search model with its tail loop excluded in the initial calculations. The model was refined using program REFMAC5^21^ in the CCP4 suite, and manually built in COOT^22^. Nucleozin was modelled into positive electron-density maps after a few rounds of restrained refinement and the stereochemistry restraints were generated using PRODRG^23^. Six NP molecules were found in the asymmetric unit and they formed two NP trimers. The two trimers were almost identical with an r.m.s.d. value less than 0.3 Å for all main chain atoms. So the NCS refinement was applied in the last round of refinement with the two timers aligned. Quality of the refined data was finally evaluated by program PROCHECK^24^ in the CCP4 suite. Data collection and refinement statistics are summarized in Table 1. Coordinates and structure factors have been deposited in the protein data bank, under PDB ID code 5B7B.

### Modelling of nucleozin induced NP aggregates

Because nucleozin induced large aggregates of NP in solution, NP-nucleozin cocrystals could not be easily obtained. To show how nucleozin induced large NP aggregates, in silicon modelling was performed based on the structures of NP-nucleozin (PDB code 5B7B) and NP-compound3 (PDB code 3RO5)^14^. Briefly, the two NP trimers in the asymmetric unit were separated and rotated in Coot. One NP molecule from trimer A was docked to one NP molecule from trimer B bottom-to-bottom using nucleozin and the two binding pockets as bridge. The detailed interaction was optimized using Rosetta docking online software^25^ (http://rosettadock.graylab.jhu.edu). With elaborate placement of the starting NP-nucleozin-NP interaction pair, the docking process can be repeated to form large aggregates.

## Additional Information

**How to cite this article**: Pang, B. *et al*. Structural Characterization of H1N1 Nucleoprotein-Nucleozin Binding Sites. *Sci. Rep.* **6**, 29684; doi: 10.1038/srep29684 (2016).

## Figures and Tables

**Table 1:** Crystallographic data collection and refinement statistics.

	*NP-nucleozin*(*PDB code 4* × *9A*)
**Data collection**	
Space group	*C2*
Cell dimensions	
a, b, c (Å)	*129.0, 121.6, 195.5*
α, β, γ (°)	*90.00, 89.79, 90.00*
Resolution (Å)	*50~3.00(3.11~3.00)*
Unique reflections	*55770(5662)*
Completeness (%)	*92.3(94.6)*
Redundancy	*3.3(3.4)*
Wilson B-factor	*71.44*
Rmerge	*0.086(0.557)*
I/σI	*16.1(1.9)*
**Refinement**	
Resolution (Å)	*50~3.00*
Rwork	*0.2001*
Rfree	*0.2623*
No. atoms	*19954*
Protein	*19894*
ligands	*60*
Protein residues	*2533*
Ramachandran plot	
Favored (%)	*95.0*
Allowed (%)	*4.7*
Outliers (%)	*0.2*
Average B-factors	*75.76*
Protein	*75.62*
ligands	*119.43*
R.m.s. deviations	
Bond lengths (Å)	*0.010*
Bond angles (°)	*1.42*

*Values for the highest resolution shell are given in parentheses.
